# Supplementary figures and images for: Identification of Novel mRNA Isoforms Associated with Acute Heat Stress Response Using RNA Sequencing Data in Sprague Dawley Rats
Source: Biology (Basel). 2022 Nov 29;11(12):1740. doi: 10.3390/biology11121740 (PMC9774719; doi:10.3390/biology11121740)

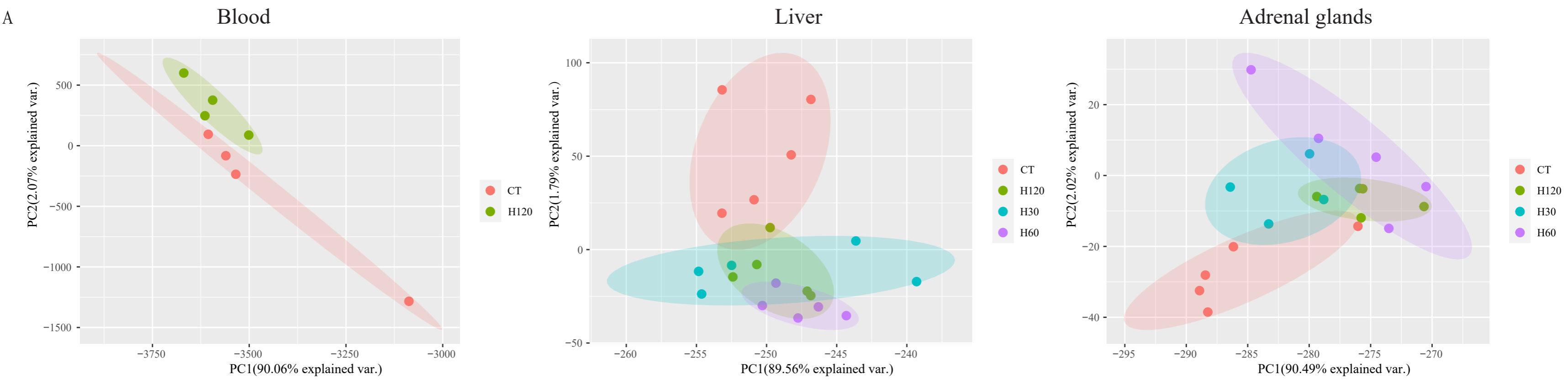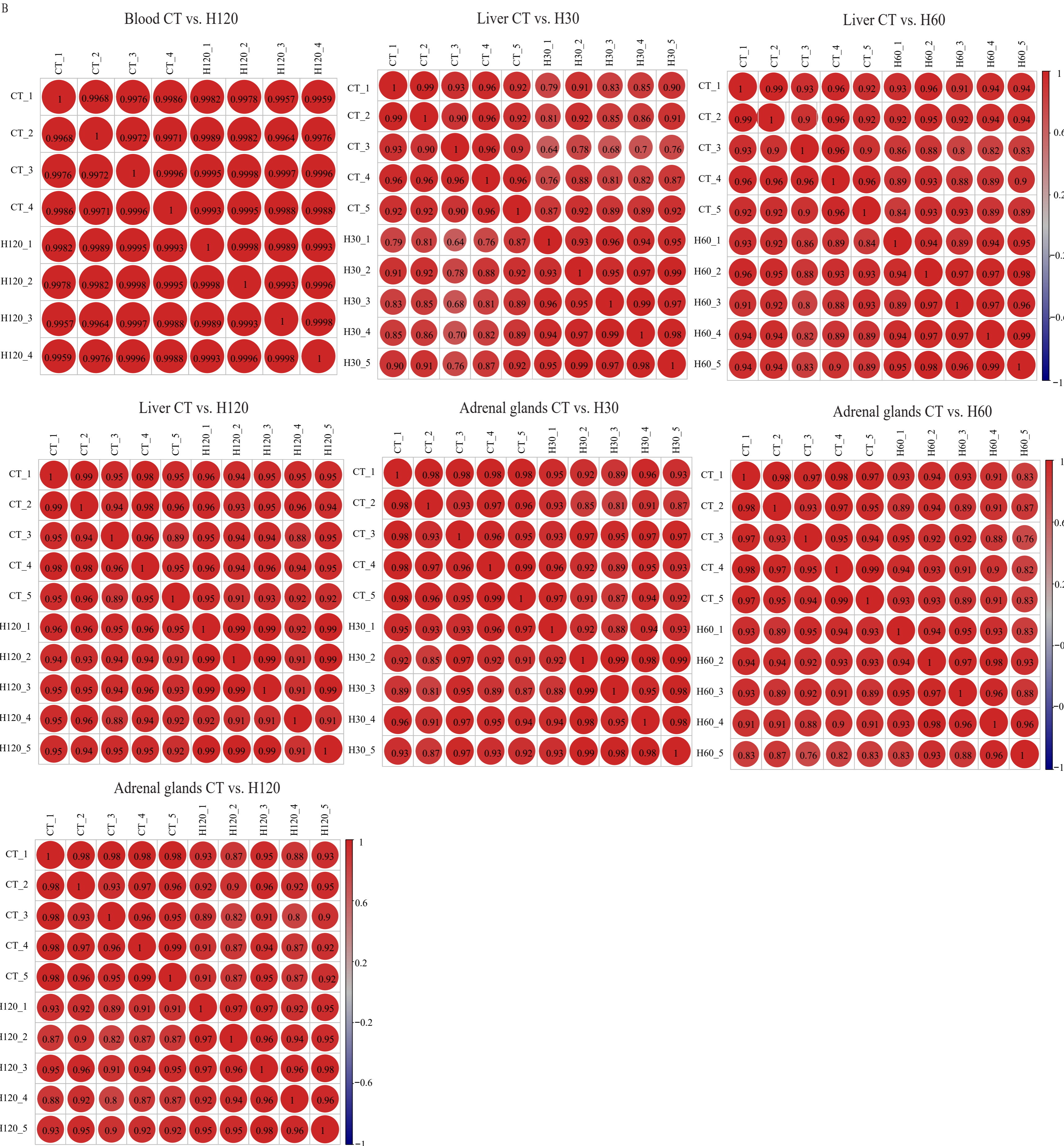

Supplement: Supplementary file 1 [file biology-11-01740-s001.zip › Figure S1.pdf]
